# The Responsiveness of Patients’ Quality of Life to Dental Caries Treatment—A Prospective Study

**DOI:** 10.1371/journal.pone.0164707

**Published:** 2016-10-24

**Authors:** Ding-Yu Yeh, Hsiao-Ching Kuo, Yi-Hsin Yang, Pei-Shan Ho

**Affiliations:** 1 Department of Dentistry, Kaohsiung Armed Forces General Hospital, Kaohsiung, Taiwan; 2 School of Dentistry, College of Dental Medicine, Kaohsiung Medical University, Kaohsiung, Taiwan; 3 School of Pharmacy, College of Pharmacy, Kaohsiung Medical University, Kaohsiung, Taiwan; 4 Department of Oral Hygiene, Kaohsiung Medical University, Kaohsiung, Taiwan; Virginia Commonwealth University, UNITED STATES

## Abstract

The objective of this study was to determine the responsiveness of oral health–related quality of life (OHRQoL) (oral health impact profile [OHIP] and oral impact on daily performance [OIDP]) and health-related quality of life (HRQoL) (World Health Organization quality of life scale, brief [WHOQOL-BREF]) in dental caries restoration treatment. The study also aimed to assess the influence of treatment on the responsiveness of patients’ quality of life (QoL). A total of 126 patients (aged 16–40 years) received dental caries restoration treatment with a 2-week follow-up and pre- and posttreatment interviews by questionnaire. Patients were assessed for their perceptions of OHRQoL and HRQoL by using the OHIP, OIDP, and WHOQOL-BREF measures. The responsiveness of all outcome measurements was assessed by effect size (ES). Stepwise multiple regression analysis was used to examine the association with the responsiveness of all outcome measurements. Significant differences were found between OIDP (ES = 0.39), OHIP (ES = 0.54), and WHOQOL-BREF (ES = 0.13) with regard to pretreatment and posttreatment (p-values: <0.0001, <0.0001, and 0.0120, respectively). Sex and dental caries status at baseline were significantly associated with responsiveness by all measurements. This study suggests that dental caries treatment moderately improves OHRQoL, but is less related to HRQoL. Furthermore, the number of dental caries and restoration are important factors affecting the improvement of patients’ perceived OHRQoL.

## Introduction

Dental caries is the most prevalent of oral diseases, affecting approximately 97% of the population during individuals’ lifetimes [1]. A perception persists among dentists that dental caries is active only in the younger population [2–4], and that the relatively low caries experience found in those over 18 years old is delayed until later in life [5]. More recently, however, reviews of cohort studies of caries in adults have found that they are caries active. Dental caries is a multifactor disease that is likely modulated by immunological, microbial, genetic, behavioral, and environmental contributors to risk, which ultimately determine the severity of clinical disease. Dental caries affects individuals’ ability to eat as well as their food choices, communication, and appearance, decreasing their quality of life. In the last 2 decades, patient satisfaction with care and quality of life (QoL) has been increasingly recognized as an important outcome of care. QoL has been described as a multidimensional concept including physical, emotional, social, and other factors [6–8]. Fazekas [9] supported a concept of total patient care: not only should basic treatment and prevention be addressed while taking care of a patient, but also the physical, psychological, and social aspects of disease and disorders. Oral conditions pertaining to problems with eating, nutrition, interaction, and emotional and psychological functions, as well as the idea that discomfort, disability, and oral impairment affect QoL, have been described by Reisine and Miller [10] and Adulyanon et al. [11]. In dentistry, oral health care is no longer merely seen as the clinical appearance of oral health conditions or the treatment of diseases. More attention has been given to how effective dental treatment can improve different aspects of patients’ lives. This attention has led to the development of instruments that measure aspects of QoL.

The perception of the oral health–related quality of life (OHRQoL) has been shown in previous studies by investigators such as Locker and Miller [12], Locker and Slade [13], McGrath et al. [14,15], John et al. [16], and Steele et al. [17] to be related to oral health status, especially the caries status. The association is especially true for the “decayed” and “missing” tooth aspect of caries. Current OHRQoL questionnaire instruments were used to assess the impact of various dental treatments on QoL, such as the Oral Health Impact Profile (OHIP-49) [18–20], OHIP-14 (short version) [15,21–26], OHRQoL [27], OHRQoL-UK [15], Children Perception Questionnaire (CPQ) [28], and the Oral Impact on Daily Performance (OIDP) [22,24,29,30]. Several studies support high internal consistency and content validity for measuring the influence of the treatments on the responsiveness of patients’ QoL, especially in OHIP-14 and OIDP. OHIP-14 has good reliability, validity, and precision, which was verified by Slade [31]. It has also been used to assess the differences between pre- and posttreatment of dental problems [25]. OIDP, which is a reliable and valid indicator of oral impact and oral satisfaction, was developed by Adulyanon et al. [11] and then applied for patients receiving orthodontic treatment [22]. Some studies have used Health-Related Quality of Life (HRQoL) questionnaires to assess the differences between pre- and posttreatment of dental problems, and the 36-item Short-Form and World Health Organization Quality of Life Scale Brief (WHOQOL-BREF) have often been used to assess prosthodontic treatment [24,32]. These two instruments have good reliability and validity.

At present, several longitudinal studies regarding OHRQoL have mainly focused on aspects of periodontal disease [27,29], prosthodontics [19,20,24,33], orthodontics [21,22], and oral surgery [15,23]. Previous periodontal treatment studies revealed improvement of OHRQoL in periodontal treatment, especially for pain, eating and chewing, and psychologic function [27], and the effect size (ES) of OHRQoL showed moderate clinical differences [27,29]. Previous studies, which used OHIP-14 [21,22] and OIDP [22] questionnaires for assessment, indicated significant improvements of OHRQoL after orthodontic treatment. McGrath et al. [15] used OHRQoL-UK and OHIP-14 scales to assess OHRQoL in patients receiving third molar extraction under local anesthesia, and only found a small improvement. Further, van Wijk et al. [23] also found third molar extraction to have an apparently good effect, but the complications had a converse effect, thus resulting in poor OHRQoL. In prosthodontic treatment, OHRQoL was significantly improved, as measured by OHIP-14 [24], OHIP-49 [19,20,33], and OIDP[24], but there were no significant differences in HRQoL based on WHOQOL. Awad et al. [33] used the OHIP-49 questionnaire to investigate patients receiving mandibular implant-supported overdentures and found significant improvement in 7 domains of OHIP-49. Also, some papers have discussed the association of caries and QoL in adults. In 2013, Espinoza et al. [34] revealed an inverse association between the number of remaining teeth and impairment in aspects of OHRQoL, which supported the findings from other studies that incremental tooth loss is an important contributor to poor OHRQoL [34]. Also, in some studies [34–36], the number of teeth with untreated caries was positively associated with impaired OHRQoL.

However, limited research has been done on how OHRQoL and HRQoL respond to dental caries restoration treatments. Therefore, the aim of this study was to assess responsiveness of OHRQoL and HRQoL in patients with dental caries restoration treatment and to determine the influence of treatment on the responsiveness of patient QoL. In brief, we examined the study hypothesis that caries treatment affects the improvement of QoL.

## Materials and Methods

### Subjects and study design

This prospective follow-up study included patients within the defined age bracket at the Department of Dentistry in the Kaohsiung Armed Forces General Hospital and in the Pingtung branch of the same institution from February to September 2011. These patients presented with self-reports of toothache, discomfort, food impaction, and so forth. They underwent dental examination and treatment, and they were found to have dental caries. A total of 135 patients received dental caries restoration treatment, and 126 of them completed questionnaires both before treatment and after treatment (approximately 2 weeks later). Nine subjects were lost to follow-up (still unavailable after telephone contact), and the follow-up rate was more than 90%.

The exclusion criteria were root caries, pulpitis after dental caries restoration, pulp capping after removal of dental caries, pulp necrosis, and incomplete or complete root canal treatment. Written informed consent was obtained from all participants. This study was approved by the Institutional Review Board, Kaohsiung Armed Forces General Hospital (100–027). We obtained written informed consent from guardians on behalf of the children enrolled in our study.

### Clinical data collection

The contents of the oral examination chart included dentition status, plaque index, and crowded incisor status at pretreatment.

The standard oral examination equipment consisted of a dental chair, dental probes, and handheld mirrors. X-ray imaging was used for diagnosis when necessary. Data collection and oral examinations were performed by one well-trained dentist. After treatment, the dentist recorded the location and surface of treated caries.

Following the study design of Oscarson et al. [26], our study defined a decayed, missing, and filled teeth (DMFT) index of more than 8 as indicative of serious dental caries, and we divided the patients into four groups based on dental caries status: DMFT index ≤ 8 and complete dental caries restoration (A group); DMFT index ≤ 8 with untreated dental caries (B group); DMFT index > 8 with complete dental caries restoration (C group); and DMFT index > 8 with untreated dental caries (D group).

### Questionnaire

Information obtained by questionnaire included demographic characteristics (sex, age group, education level, and economic status), time of replacing toothbrush, perceived oral and general health condition, and OIDP, OHIP, and WHOQOL-BREF measurements.

### Oral health–related quality of life

We used two questionnaires to collect OHRQoL status, including OHIP-14T (Taiwan version) from Kuo et al. [37] and the OIDP. For each OHIP item, subjects were asked how frequently they had experienced an oral health impact from a particular item during the last 12 months. Responses to OHIP questions were made on a 5-point Likert scale based on how frequently the problem occurred: 4 = very often; 3 = often; 2 = occasionally; 1 = rarely; and 0 = never. The total OHIP score was the sum of all the individual item scores for the 14 questions. The OHIP is divided into seven dimensions (functional limitation, pain, psychological discomfort, physical disability, psychological disability, social disability, and handicap) represented by the sum of the item scores within each conceptual dimension. Lower OHIP scores indicate better OHRQoL.

The OIDP instrument was used to assess the oral impact on daily life in relation to nine items: eating, speaking, cleaning teeth, physical activities, sleeping, smiling, emotional stability, major role activity, and contact with people [11]. Each performance score of OIDP was calculated by multiplying frequency (0–5) and severity scores (0–5). These scores for the nine performances were then summed up. The overall OIDP score was the sum divided by the maximum possible score (225) and multiplied by 100 to give a percentage score. Lower OIDP scores indicate better OHRQoL.

### Health-related quality of life

The HRQoL for each subject was measured by the Taiwanese version of WHOQOL-BREF [38]. The 28 items in this questionnaire include two general items, 24 items universally adopted for WHOQOL-BREF to cover four dimensions (physical, psychological, social, and environment), and two national items that were more specific to the culture of people in Taiwan. The scale of the WHOQOL-BREF consisted of frequency, intensity, capability, and evaluation in a 5-point response format (scored from 1 to 5). Each dimension score was calculated by multiplying the mean of all facet scores in the same domain by a factor of 4, with a higher score indicating a better HRQoL (range 4–20).

### Data analysis

The statistical analyses were carried out by SPSS 19.0. Internal consistency (reliability) was evaluated for all measurements (OHIP, OIDP, and WHOQOL-BREF) by Cronbach’s alpha. The criterion-related validity evaluated the association between all measurements and patients’ perceived oral and general health condition by one-way ANOVA test. Paired *t-*test was used to evaluate the responsiveness of all measurements with dental caries treatment. Cohen’s standardized ES [39], was computed to evaluate the responsiveness of different measurements, which could be considered as several levels of clinical meaningfulness (small, 0.2 ≤ ES < 0.5; moderate, 0.5 ≤ ES < 0.8; and large, 0.8 ≤ ES).

Stepwise multiple regression analysis (an entry probability of 0.05 and a removal probability of 0.05) was used to assess the association with responsiveness for all measurements, while adjusting for the pretreatment score.

## Results

A total of 135 patients were invited to participate in the study, and 126 patients completed both pre- and posttreatment questionnaires. Table 1 shows the demographic characteristics at pretreatment. Of the participants, 79.3% were male, 20.7% were female, and 76.30% were 20 years of age and older. More than half of the subjects (52.59%) were college graduates or above, and 49.63% of subjects had a economic status that was sufficient for daily life. Table 2 shows oral examination status, oral health behavior, and treatment status. For dental caries status, DMFT index > 8 and complete dental caries restoration (C group) was 36.51%, the plaque index mean was 0.64, and the maximum plaque index was 0.97. Crowded incisor status was 25.19%, and the proportion of participants who replaced their toothbrushes within 3 months was 47.41%. The mean number of teeth treated for caries was 2.51. A total of 126 participants received questionnaire interviews before and after treatment, yielding a response rate of 93.33%.

**Table 1:** Demographic characteristics at pretreatment for 135 subjects.

Variables		n	(%)
Sex			
	Male	107	(79.26)
	Female	28	(20.74)
Age group			
	16–19 years	32	(23.70)
	≥20 years	103	(76.30)
Education level			
	Junior high school and below	10	(7.41)
	Senior high school	54	(40.00)
	College and above	71	(52.59)
Economic status			
	Enough	67	(49.63)
	Just enough	43	(31.85)
	Insufficient	25	(18.52)

**Table 2:** The distribution of oral examination status, oral health behavior and treatment status (N = 135).

Variables			
Dental caries status, n (%)^**#**^^**†**^			
	A group	31	(24.60)
	B group	13	(10.32)
	C group	46	(36.51)
	D group	36	(28.57)
Mean of plaque index (SD)			0.64 (0.63)
Maximum of plaque index, mean (SD)			0.97 (0.85)
Crowded incisor status, n (%)			
	No	101	(74.81)
	Yes	34	(25.19)
Timing of replacing toothbrush, n (%)			
	Within 3 months	64	(47.41)
	4–6 months	39	(28.89)
	Over 7 months	32	(23.70)
**No. of treated caries teeth, n (%)**^**#**^			2.51 (1.38)

^#^Due to missing data, case numbers of each item may not sum up to total.

^†^Dental caries status: DMFT index ≤ 8 and complete dental caries restoration (A group), DMFT ≤ 8 with untreated dental caries (B group), DMFT > 8 with complete dental caries restoration (C group), and DMFT > 8 with untreated dental caries (D group).

For the internal consistency of the three measurements (not shown in table), the Cronbach’s alpha of the OHIP and the seven domains were 0.95 and 0.72–0.88, respectively; the functional limitation domain was the lowest; the psychological disability and social disability domains were the highest; and the OIDP was 0.94. The WHOQOL-BREF and the four domains were 0.92 and 0.64–0.80, respectively, with the social domain at the lowest (Cronbach’s alpha: 0.64) and the environmental domain at the highest (Cronbach’s alpha: 0.80). Therefore, the OIDP, OHIP, and WHOQOL-BREF measurements all had good reliability.

The discriminatory ability was evaluated by comparing the mean scores of all measurements among groups of very good, poor, or very poor in perceived oral/general health conditions (Table 3). The mean scores of the OHIP and OIDP scores were significantly increased from very good to very poor (p < 0.0001 and p = 0.0130) in perceived oral health conditions. Patient-reported general health condition was not significantly associated with the OHIP and OIDP measurements (p = 0.1570, p = 0.4180), but it was significantly associated with the WHOQOL-BREF measurement (p < 0.0001).

**Table 3:** Discriminatory ability of all measurements in perceived oral and general health condition.

			OHIP		OIDP		WHOQOL-BREF	
Variables		n (%)	Mean (SD)	p-value	Mean (SD)	p-value	Mean (SD)	p-value
Perceived oral health conditions								
	Very good/good/fair	83 (61.5)	26.48 (14.98)	<0.0001	9.40 (12.96)	0.013	62.45 (8.38)	0.156
	Poor	42 (31.1)	33.90 (14.12)		9.48 (8.19)		60.28 (7.13)	
	Very poor	10 (7.4)	44.10 (11.54)		21.20 (16.20)		58.38 (6.12)	
Perceived general health conditions								
	Very good/good	48 (35.6)	27.46 (15.02)	0.157	9.84 (10.94)	0.418	66.36 (6.55)	<0.0001
	Fair	73 (54.0)	30.67 (15.14)		9.81 (12.30)		59.37 (7.29)	
	Poor/very poor	14 (10.4)	36.14 (16.35)		14.41 (16.17)		55.68 (6.96)	

Responsiveness for all measurements assessed on dental caries treatment are presented in Table 4. OIDP total score showed a mean of 5.65 (±10.24) in posttreatment, which was significantly lower than the score of 10.51 (±12.40) (p < 0.0001) in pretreatment; the ES was 0.39. Similarly, for the OHIP total scores, the mean score of the posttreatment was 22.25 (±15.00), which was significantly lower than the pretreatment score of 30.54 (±15.33) (p < 0.0001), with the ES being 0.54. The ES of the seven OHIP domains ranged from 0.33 to 0.78. In particular, the ES of the physical pain domain was the highest at 0.78, and the psychological discomfort domain was second at 0.63. In terms of WHOQOL-BREF total scores, the mean score of the posttreatment (62.35 ± 8.16) was significantly higher than the pretreatment (61.32 ± 7.95) (p = 0.0123), with the ES being only 0.13. The four domains showed no significant change in pre- or posttreatment, with the ES being 0.04–0.11.

**Table 4:** Responsiveness of all measurements assessed in 126 subjects with dental caries treatment.

	Pretreatment	Posttreatment	Observed effect	Paired-*t* test	Effect size (ES)
Dimension	Mean (SD)	Mean (SD)	Mean (SD)	p-value	
OIDP score	10.51 (12.40)	5.65 (10.24)	4.86 (11.70)	<0.0001	0.39
OHIP score	30.54 (15.33)	22.25 (15.00)	8.29 (14.25)	<0.0001	0.54
Function limitation	5.60 (2.40)	4.48 (2.82)	1.12 (2.39)	<0.0001	0.47
Physical pain	7.76 (3.91)	5.88 (3.77)	1.88 (3.54)	<0.0001	0.78
Psychological discomfort	5.95 (2.72)	4.25 (2.81)	1.70 (2.55)	<0.0001	0.63
Physical disability	4.15 (3.16)	3.11 (2.72)	1.04 (2.94)	<0.0001	0.33
Psychological disability	3.11 (2.92)	2.02 (2.51)	1.09 (2.65)	<0.0001	0.37
Social disability	1.75 (1.86)	1.09 (1.51)	0.66 (1.67)	<0.0001	0.35
Handicap	2.21 (2.41)	1.42 (1.84)	0.79 (2.46)	0.0004	0.33
WHOQOL-BREF score	61.32 (7.95)	62.35 (8.16)	1.03 (4.53)	0.0123	0.13
Physical	14.04 (2.24)	14.29 (2.32)	0.25 (1.48)	0.0556	0.11
Psychological	13.79 (2.19)	13.95 (2.24)	0.16 (1.57)	0.2432	0.07
Social	13.76 (2.17)	13.98 (2.04)	0.22 (1.43)	0.0837	0.10
Environmental	13.31 (2.11)	13.39 (2.02)	0.08 (1.46)	0.5170	0.04

Our study used stepwise multiple regression analysis to determine the important factors related to responsiveness of all measurements (OHIP, OIDP, and WHOQOL-BREF) while adjusting for the pretreatment measurement score (Table 5). In terms of the OHIP outcome variable, sex, dental caries status, maximum plaque index, time for replacing toothbrush, and number of teeth treated for caries were selected and significantly associated with responsiveness of the OHIP. The R^2^ value of this model was 0.46. For OIDP, sex, dental caries status, and mean plaque index were selected and significantly associated with responsiveness of the OIDP. The R^2^ value of this model was 0.52. In terms of the WHOQOL-BREF outcome variable, sex, dental caries status, and crowded incisor teeth were selected and significantly associated with responsiveness of the WHOQOL-BREF. The R^2^ value of this model was 0.21.

**Table 5:** The association with responsiveness of all measurements for patients with dental caries treatment.

		OHIP^#^			OIDP^#^			WHOQOL-BREF^#^		
Variables		β-value	(95% CI)	p-value	β-value	(95% CI)	p-value	β-value	(95% CI)	p-value
**Sex**										
	Female	Reference			Reference			Reference		
	Male	−9.26	(−14.96, −3.55)	0.0017	−5.47	(−9.47,−1.46)	0.0079	−2.07	(−3.88, −0.26)	0.0256
**Dental caries status**^**†**^										
	A group	7.41	(1.71, 13.11)	0.011	7.47	(3.22, 11.71)	0.001	2.79	(0.73, 4.83)	0.008
	B group	3.16	(−3.96, 10.28)	0.382	5.63	(0.21, 11.05)	0.042	1.97	(−0.66, 4.61)	0.141
	C group	3.96	(−1.01, 8.94)	0.117	5.85	(2.09, 9.61)	0.003	1.27	(−0.59, 3.14)	0.179
	D group	Reference			Reference			Reference		
**No. of treated caries teeth**		-1.7	(−3.29, −0.12)	0.0354						
R^2^			0.46			0.52			0.21	

^#^ Analysis models were also adjusted by the pretreatment measurement score, mean of plaque index, maximum of plaque index, crowded incisor teeth, time of replacing toothbrush

^†^Dental caries status: DMFT index ≤ 8 and complete dental caries restoration (A group), DMFT ≤ 8 with untreated dental caries (B group), DMFT > 8 with complete dental caries restoration (C group), and DMFT > 8 with untreated dental caries (D group).

## Discussion

Limited studies have prospectively followed the responsiveness of OHRQoL and HRQoL to caries treatment in adults. Dental caries is not only a problem in childhood but also occurs at a relatively constant rate throughout life [40]. In our study, we found that caries treatment can improve the OHRQoL, but fewer improvements were found in HRQoLs. Our study had several advantages. First, all dental examinations were undertaken by a well-trained dentist, so the validity of dental status is quite high. Second, we used several QoL questionnaires that include the dimension of oral health–related status and overall health, and we verified the responsiveness of the different QoL scales. All three questionnaires used in this study had good reliability, and Cronbach's alpha was 0.95, 0.94, and 0.92 for OHIP, OIDP, and WHOQOL-BREF, respectively. Compared with other studies of dental treatment [15,21,22,24,25,27], our questionnaires can be used as a stable tool to investigate OHRQoL and HRQoL of patients receiving dental caries treatment.

Many studies [24,27,41] of dental intervention have used Cohen’s standardized effect [42] as a means of identifying differences that are likely to be clinically meaningful. In our study, the responsiveness of dental caries treatment was shown by ES, which was 0.54, 0.39, and 0.13 for OHIP, OIDP, and WHOQOL-BREF, respectively. The ES showed significant improvement after dental caries restoration intervention, similar to periodontal treatment, which can reduce pain and discomfort. Saito et al. [27] found that the effect size for OHRQoL change scores were moderate (ES = 0.51), and significant improvement was observed after periodontal treatment. In the study by Barbosa et al. [28], the treatment of temporomandibular disorder yielded an ES of 0.62, which was moderate improvement. In a study by Berretin-Felix et al. [24] on prosthodontics with implant treatment, both OHIP-14 and OIDP showed significant improvement after treatment, but no statistical improvement was seen with the WHOQOL-BREF scale. Our results yielded a similar finding; dental caries treatment intervention moderately improved the OHRQoL, but it had almost no effect on improvement of HRQoL. Moreover, Mashoto et al. [30] calculated an ES of 0.2 after tooth extraction or traumatic restorative treatment, showing a small degree of improvement. They showed these dental interventions were associated with a small improvement in OHRQoL. Locker et al. [25] sampled general dentistry patients for treatment and also found a small degree of improvement of 0.32 in the OHIP-14 scale for OHRQoL. Therefore, we confirmed that the OHRQoL (OHIP and OIDP) is sensitive to dental caries treatment, but HRQoL (WHOQOL-BREF) only shows a slight response. In previous studies, OHRQoL for patients undergoing serious dental procedures (e.g., extraction, surgery) had better improvement than those receiving noninvasive treatment (e.g., scaling, restoration). OHRQoL was inconsistent in previous studies, in which the improvement magnitude of OHRQoL after different dental treatment intervention was measured. HRQoL showed limited improvement in oral diseases treatment.

In the present study, only ES in the overall OHIP showed moderate improvement. Two dimensions of OHIP, physical pain and psychological discomfort, showed the most significant improvement, and the ES was 0.78 and 0.63, respectively. In previous studies of OHIP improvement in complete denture intervention, physical pain and psychological discomfort were the most common dimensions with significant improvement [43–45]. Eating was previously the most improved function reported with regard OHRQoL [46]. Physical pain and psychological discomfort of OHRQoL would be the expected dimension because the mouth is directly involved in chewing and biting, and thus enjoyment of eating.

Our finding of an inverse association between the number of remaining teeth and impairment in aspects of OHRQoL support the finding from other studies that incremental tooth loss is an important contributor to poor OHRQoL. Another important factor affecting QoL is dental caries status, as shown by group with DMFT index ≤ 8 and complete dental caries restoration having the most significant improvement in QoL. Similar results were also found by Broder et al. [47], Biazevic et al. [48], and Cohen-Carneiro et al. [49]. Some previous studies [34–36] also showed that the number of teeth with untreated caries was positively associated with impaired OHRQoL. The other possible reason might be that a subject with a high DMFT index tends to have less ability to undertake oral care. In addition, other dental caries problems may coexist with untreated dental caries and cause toothache and discomfort, thus affecting quality of life. This study has limitations that are worthy of follow-up. Scales such as OHIP, OIDP, and WHOQOL-BREF all require a patient to recall events 2 weeks, 6 months, and 1 year in the pretest, and 2 weeks after treatment in the posttest, thus possibly overestimating the severity with regard to QoL. However, caries treatment is different from other dental therapy (e.g., orthodontic, periodontic, oral surgery and prosthodontic therapy) [50]. In general, caries treatment only requires one visit and fewer patients complain of any symptoms after the placement of the composite resin. Direct composite restorations is the most common caries treatment, and the tooth-filling preservation rate for 2 years is 91.79%–93.20% in Taiwan 2012. Furthermore, the subjects in this study were mainly servicemen and their family members. The results we obtained cannot be extrapolated to other dental caries patients in other age groups. Therefore, further study is necessary. In addition, we can neither differentiate between nor make any record of the degree of dental caries severity in this study because clinical degrees of dental caries severity can only be judged from the treatment methods (e.g., restoration, pulp capping, endodontic treatment, extraction). As for the patient’s chief complaint, we could not evaluate any specific degree of pain caused by dental caries. Consequently, an apparent difference is likely made in the evaluation of life quality improvement.

## Conclusion

The OHRQoL scale showed significant responsiveness in patients receiving restoration treatment for dental caries, but on the HRQoL scale, patients showed only a slight improvement.

Through follow-up observations on treatment of our dental patients, dentists can not only help patients eliminate their oral diseases, but they can also take care of their physical, psychological, and social needs, in line with practicing total patient care. Our findings also prove that restoration treatment for dental caries is an important contributor to improving OHRQoL.

## Supporting Information

S1 File data_plos one.xlsx (XLSX)

## Abbreviations

CPQ: Children Perception Questionnaire
DMFT: Decayed, Missing, and Filled Teeth
ES: Effect Size
HRQoL: Health-Related Quality of Life
OHIP: Oral Health Impact Profile
OHRQoL: Oral Health–Related Quality of Life
OIDP: Oral Impact on Daily Performance
QoL: Quality of life
WHOQOL-BREF: World Health Organization Quality of Life Scale Brief
